# Sole coloration as an unusual aposematic signal in a Neotropical toad

**DOI:** 10.1038/s41598-018-37705-1

**Published:** 2019-02-04

**Authors:** Daniela C. Rößler, Stefan Lötters, Johanna Mappes, Janne K. Valkonen, Marcelo Menin, Albertina P. Lima, Heike Pröhl

**Affiliations:** 1 Department of Biogeography, Trier University, Universitätsring 15, 54296 Trier, Germany; 2 Centre of Excellence in Biological Interactions, Department of Biological and Environmental Science, University of Jyväskylä, P.O. Box 35, 40014 Jyväskylä, Finland; 3 Department of Biology, Institute of Biological Sciences, Amazonas Federal University, Av. General Rodrigo Otávio Jordão Ramos 3000, 69077-000 Manaus, Brazil; 4 Coordenação de Pesquisas em Biodiversidade, Instituto Nacional de Pesquisas da Amazônia, Av. André Araujo 2936, 69011–970 Manaus, Brazil; 5 Institute of Zoology, University of Veterinary Medicine Hannover, Bünteweg 17, 30559 Hannover, Germany

**Keywords:** Behavioural ecology, Evolutionary ecology

## Abstract

Many animals have evolved remarkable strategies to avoid predation. In diurnal, toxic harlequin toads (*Atelopus*) from the Amazon basin, we find a unique colour signal. Some *Atelopus* populations have striking red soles of the hands and feet, visible only when walking. When stationary, the toads are hard to detect despite their yellow-black dorsal coloration. Consequently, they switch between high and low conspicuousness. Interestingly, some populations lack the extra colour display of the soles. We found comprehensive support that the red coloration can act as an aposematic signal directed towards potential predators: red soles are significantly more conspicuous than soles lacking red coloration to avian predators and the presence of the red signal significantly increases detection. Further, toads with red soles show bolder behaviour by using higher sites in the vegetation than those lacking this signal. Field experiments hint at a lower attack risk for clay models with red soles than for those lacking the signal, in a population where the red soles naturally occur. We suggest that the absence of the signal may be explained by a higher overall attack risk or potential differences of predator community structure between populations.

## Introduction

Animals use diverse behavioural and physiological strategies to avoid predation. One anti-predator strategy is aposematism, in which animals combine secondary defences such as toxins with signals that inform potential predators about their noxiousness^1–3^. Aposematism is efficient when predators learn to avoid conspicuous signals as a cue of defence. Aposematic signals are known from a wide range of species and can target olfactory, acoustic, chemical, or visual senses of the predator^3–6^. In the case of aposematic coloration, potential prey display bright colours, often red, orange or yellow, forming a strong contrast against the background^7–9^. Well-known examples of aposematic coloration are the yellow and black colours of wasps^6^, the red and black colours of ladybirds^10^ as well as the striking red, white, black and yellow colours of coral snakes^11–13^.

Although the benefit of signal conspicuousness for avoidance learning has been shown in numerous experiments, many defended animals are not overly conspicuous^14,15^. Indeed, warning colours are one extreme of a continuum between crypsis and conspicuousness^16^. The efficiency of the strategy strongly depends on the respective environment. Conspicuousness depends on the background colour, complexity and the contrast that the animal’s colour provides. The benefit of conspicuousness, however, strongly depends on the predator. For example, in environments with naïve or immune predators or a high abundance of predators, aposematic colours can increase the risk of detection and consequently the risk of attack. For that reason, it may be advantageous to be less conspicuous. However, crypsis using disruptive coloration or background matching, loses its effect as soon as the animal moves^17^. A fair compromise is the combination of being aposematic and cryptic at the same time^18^. This has been shown, e.g. for the larva of the swallowtail butterfly (*Papilio machaon*)^19^. The caterpillars are cryptic from a distance and aposematic during close encounter. The alder moth (*Acronicta alni*) changes its appearance from resembling bird droppings to a striking yellow-black colour during its last instar, when moving to the pupation sites^20^. Here, it has been suggested that being aposematic during active movement can be beneficial, because these caterpillars are chemically defended.

**Figure 1:**
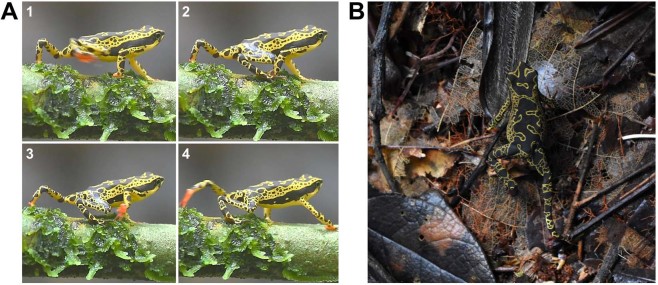
*Atelopus spumarius* sensu lato, (**A**) display of red hand and foot soles during walking; (**B**) potential disruptive dorsal coloration on a complex background. Photos: Daniela C. Rößler.

While studying Neotropical harlequin toads (*Atelopus spumarius* sensu lato) we found a unique colour signal that drew our attention. During movement, some populations of these toads display bright red foot soles which become a highly conspicuous signal (see Fig. 1A, Supplementary Video S1). Yet, dorsal patterns are complex and highly variable. They most likely function as disruptive coloration, breaking up the body outline. Despite mostly being yellow-green and black, the dorsal pattern makes it hard to detect the toads on complex backgrounds^21^ (see Fig. 1B). The following observations are noteworthy: firstly, harlequin toads are slow walkers rather than leapers^22^; secondly, *Atelopus* skin secretions contain the potent tetrodotoxin and its derivatives^23^, substances known as effective defence mechanisms in a wide array of aquatic and terrestrial animals including amphibians^24,25^. Lastly, an unken reflex behaviour is undescribed in the genus *Atelopus*. According to our own (unpubl.) observations, an unken behaviour cannot be triggered in our study species. Therefore, the function of the bright coloration remains puzzling.

We hypothesized that red feet found in *A. spumarius* sensu lato populations fulfill an aposematic function towards predators. In our study, we investigate conspicuousness, detectability, behaviour as well as predation risk. As a result, we suggest that the function of the remarkable red hand and foot soles (from here on only referred to as red feet) is a warning signal.

Within this framework, we predicted that a) the red feet of *A. spumarius* sensu lato are significantly more conspicuous to several potential predators than the feet of conspecifics lacking red coloration. To test this, we measured the reflectance of the feet in several studied populations and used subsequent visual modelling to analyse their conspicuousness to birds, snakes and crabs, all of which are important predators of frogs and toads^26–28^. Our second prediction was that b) detection rates are higher for red footed toads than for toads without red feet. This was tested with a detection experiment using clay models and human subjects as predators (from here on referred to as human predators). Our third prediction was that c) red footed toads show bolder behaviour as they should be protected if the red feet function as a successful aposematic signal. By observing their behaviour in the field, we found perch height to be an indicator of boldness behaviour, as climbing up higher positions probably entails a higher and longer risk of detectability and attack by potential predators. Especially, as they are not able to jump up on vegetation, but rather have to use unvegetated perches to slowly climb upwards, the level of exposure should increase when moving on exposed surfaces. Lastly, we predicted that d) presence of red feet translates to lowered predation risk. Predation risk was assessed by exposing clay models to natural predators in the field.

## Results

### Visual modelling

We used visual modelling to calculate colour and brightness contrast between the feet of the toads and their background (foliage as well as dorsal dark coloration). Higher contrast indicates higher conspicuousness in the eyes of the respective predator.

Pairwise comparisons indicated that the chromatic contrast of red foot coloration against a common background, as well as red feet against the dorsal dark coloration, is more perceptible than the non-red foot coloration, particularly to avian predators. The snake and crab models did not reveal any importance in chromatic contrasts. Although snake and crab models can distinguish the red feet from the background achromatically, the contrast of red feet to dorsal dark coloration is less than the contrast of non-red feet against the same background (Supplementary Table S1).

For bird eyes, the colour contrast of the red feet was significantly higher (16.14 ± 7.98 JND, N = 32) than the contrast of non-red feet (5.45 ± 2.83 JND, N = 20) against foliage (*U* = 31, p < 0.001 two-tailed). The same holds true for the contrast of red feet (17.17 ± 7.52 JND, N = 32) and non-red feet (7.63 ± 3.58 JND, N = 20) against dorsal dark colour of the toad (*U* = 66, p < 0.001 two-tailed). However, the brightness contrast did not differ between red feet and non-red feet against both substrates.

For snakes, the visual model did not reveal any significance in terms of chromaticity between red and non-red feet. But, in terms of achromatic information, for the snake the contrast of red feet (11.10 ± 7.94 JND, N = 32) was significantly higher than the contrast of non-red feet (20.86 ± 10.89 JND, N = 20) against foliage background (*U* = 149, p < 0.001 two-tailed). Against dorsal dark coloration, the achromatic contrast of red feet (11.10 ± 7.94 JND, N = 32) was significantly smaller than for non-red feet (20.86 ± 10.89 JND, N = 20) (*U* = 148, p < 0.001 two-tailed).

The chromatic contrast against foliage did not differ between the groups for crab vision. Similar to snake vision, we find a significant difference in terms of achromatic contrast between red feet (12.05 ± 3.66 JND, N = 32) and non-red feet (8.33 ± 3.70 JND, N = 20) against a foliage background (*U = *149, p < 0.001 two-tailed) with red feet showing higher contrast values. Again, we find reverse values concerning red feet (3.16 ± 2.09 JND, N = 32) and non-red feet (7.53 ± 3.89 JND, N = 20) against the dorsal dark coloration of the toad (*U* = 110, p < 0.001 two-tailed). Here, red feet show less contrast than non-red feet.

### Detectability

**Figure 2:**
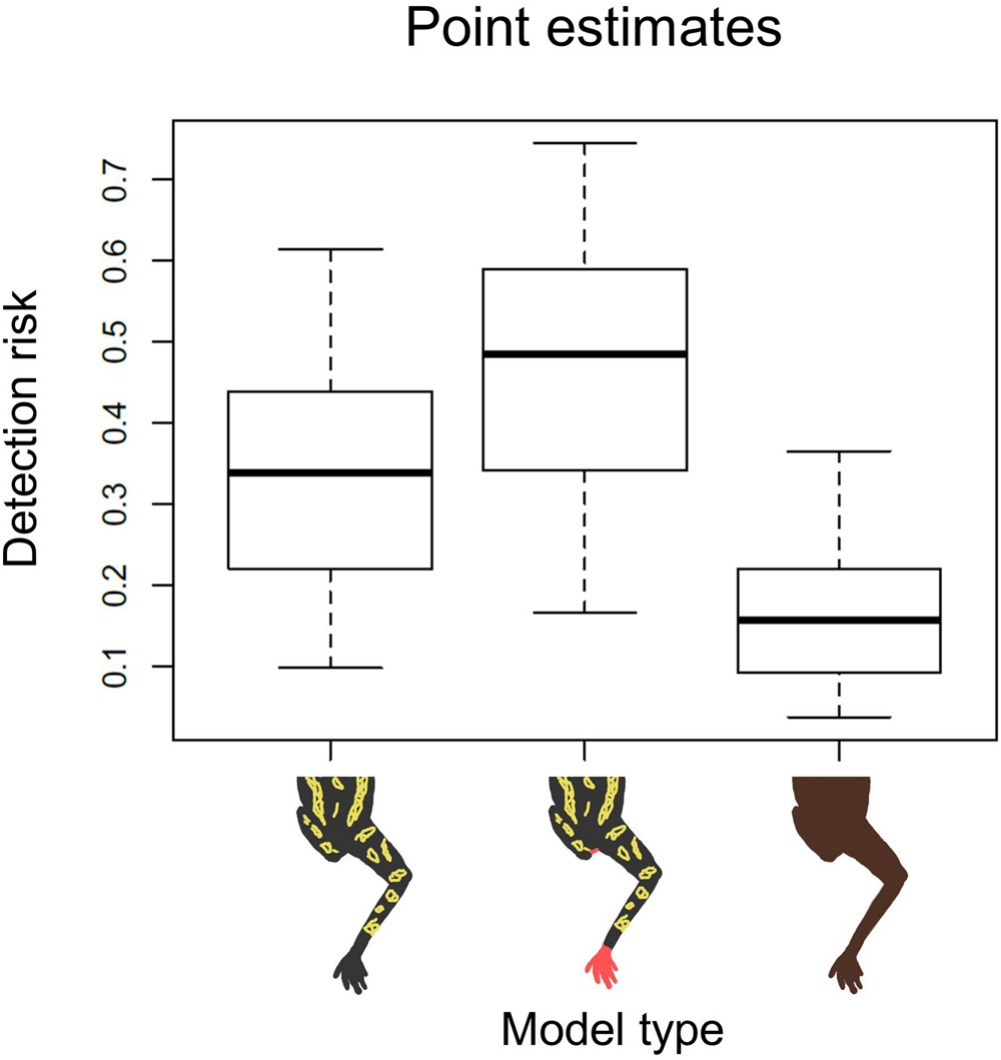
Point estimates of detection risk for non-red footed, red footed and brown models.

On average, a human predator found 12 out of 36 potential clay models (3 different morphs, 12 each). Among the detected, 6 red footed models (50%), 4 non-red footed models (36%) and 2 brown models (14%). The detection of different morphs varied significantly (Table 1, Fig. 2). Risk to be detected was higher for red footed models that to non-red footed and brown models. Brown models were also less likely detected than non-red footed models.

**Table 1:** GLMM estimating detection probabilities of artificial toads.

Random effects	Variance	Standard deviation		
Participant ID	0.54	0.74		
**Fixed effects**	**Estimate**	**Se**	**Z**	**p-value**
(Intercept) Toads without red soles	−0.78	0.21	−3.82	<0.001
Toads with red soles	0.61	0.19	3.28	0.001
Brown toads	−1.02	0.25	−4.74	<0.001

Toads without red soles on their feet are included in the intercept.

### Position heights

**Figure 3:**
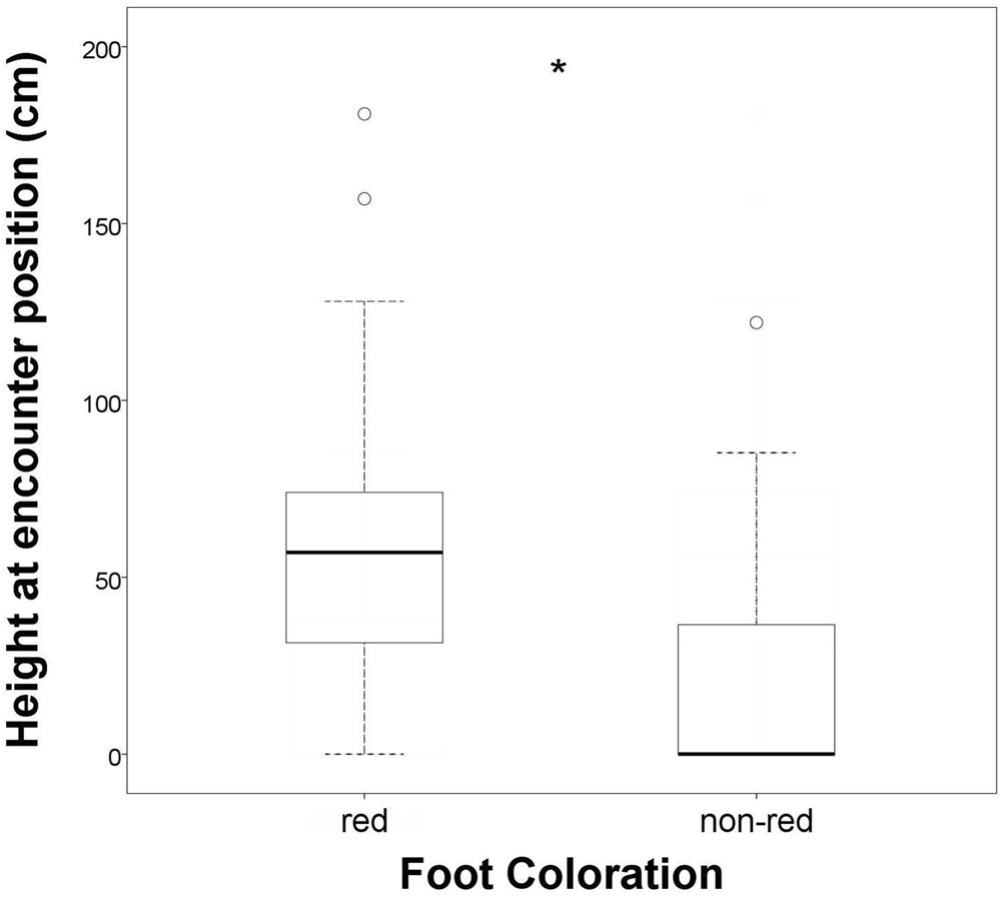
Boxplots showing position height at time of encounter for 57 individuals from a population with red feet and 54 individuals from a non-red feet population. Whiskers represent 1.5 IQR distance from the first and third quartile. *Significant.

The position height at the time of the encounter for male individuals in the population with red feet (58.1 ± 39.9 cm, N = 57) was significantly higher (*U* = 2382, p < 0.001 two-tailed, Fig. 3) than the position height of individuals in the population without the trait (21.27 ± 30.07 cm, N = 54).

### Predation experiment

**Figure 4:**
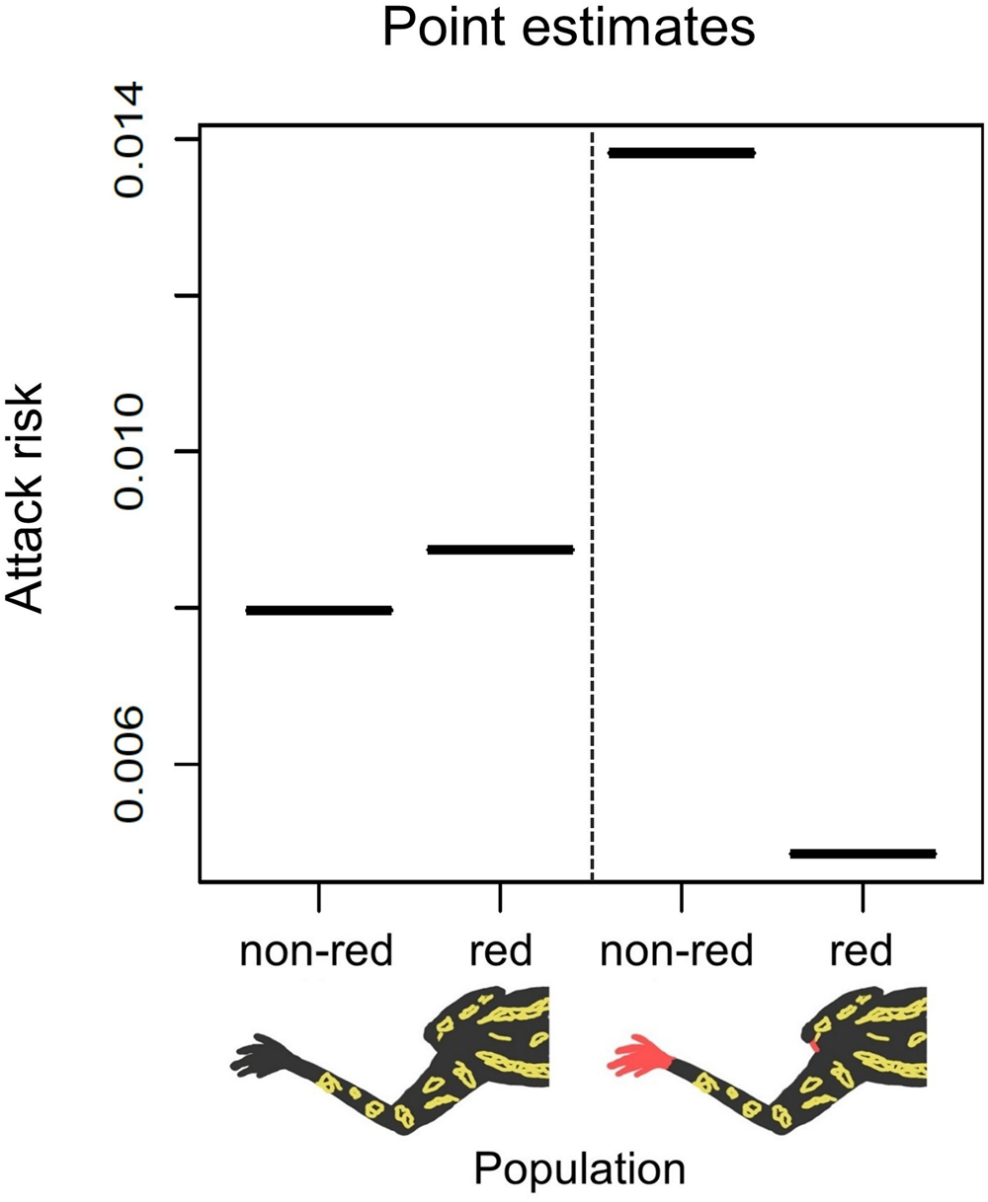
Point estimates of attack risk for red and non-red footed models in two populations.

Of the 180 models deployed, 21 were attacked by birds (raw experimental data can be found in the Supplementary Table S1). Even though we did not find significant effects of coloration or population on predation, the estimated predation risk appeared to be almost three times higher for non-red footed toads than red footed toads in the population naturally containing red footed toads (Odds ratio (OR) = 2.87). Whereas in population where red footed toads do not naturally exist, predation risk appeared to be slightly higher for red footed than non-red footed toads (OR 1.09; Table 2; Fig. 4). Furthermore, estimated attack risk for red footed toads was nearly two-fold in population without naturally occurring red footed toads than in population where they are present (OR 1.81). This suggest that local predators may have learned to avoid naturally occurring red footed toads, whereas in a population where most of the predators are not educated, they suffer elevated costs of increased detectability.

**Table 2:** GLM estimating attack probabilities of artificial toads. Factor levels population 1 and toad models without red soles are included in the intercept.

Significance of terms in the model	Df	LRT	p-value
Population	1	0.05	0.82
Sole coloration	1	1.35	0.25
Sole coloration * Population	1	1.53	0.22
**Coefficients**	**Estimate**	**se**	
(Intercept) toads without red soles in population 1			
Population 2	0.56	0.60	
Red soles	0.09	0.67	
Red soles * Population 2	−1.15	0.95

## Discussion

Predation is one of the strongest selective forces in nature that has shaped a range of remarkable anti-predator strategies. Animals displaying bright colours to inform predators about their defences, is a prime example^29^. Harlequin toads, which possess the potent tetrodotoxin (TTX) in their skin, display a variety of coloration and show highly interesting behaviour. Many populations of these diurnal toads display striking red soles of hands and feet whilst undisturbedly walking. With our study, we are the first to investigate the underlying function of this coloration. We found that red soles are significantly more conspicuous than feet lacking the coloration to avian predators and that presence of the signal significantly increases detection (by human predators). Further, we found evidence that red footed toads behave more boldly than those lacking the coloration. Additionally, field experiments hint at a lower attack risk for clay models with red soles than for those lacking the signal, in the population where the red soles naturally occur. Our results support our hypothesis that the red feet are a form of warning coloration directed towards visually oriented predators, most likely towards birds.

The visual modelling showed that red soles are conspicuous to birds, but less so towards snakes and crabs. This makes sense, because snakes likely use different sensory modalities (olfactory and infrared cues) to detect their prey^30^. Indeed, our detection experiment revealed that red feet increased the detection rate of toads by 14%. Although human and avian vision have differences, humans can be used as a proxy as they have been shown to share similarities with birds not only in vision but also in object recognition^31,32^. Moreover, the detectability estimate may be conservative, because the models in the experiment were immobile and detectability of red feet can be expected to be higher for moving prey. Interestingly, we observed behavioural differences between red footed and non-red footed populations. Toads with red soles behaved more boldly and were found higher in the vegetation than non-red footed toads. Toads that climb higher and use higher sites in the habitat are likely to become more visible to predators. Not only because the active movement on the way up increases detection risk, but also because perches used for climbing up are less vegetated and thus toads are more exposed. Similar observations are made in aposematic (conspicuous) poison frogs (*Oophaga pumilio* and *O. granulifera*) which move, forage or call more or more openly, showing bolder behaviour than respective cryptic morphs^16,33,34^. Perch height is also associated with riskier calling sites used by conspicuous *O. pumilio* populations^35^, and correlated with male mating success^36^. Although mating success is less likely to play a role in perch height choice in *Atelopus* species, they might be able to gain territorial benefits from higher sites (i.e. can oversee larger territories, which can be beneficial in spotting intruders, but also potentially females).

Conspicuous prey can suffer increased predation risk caused by its high detectability, particularly if the majority of the predator population is naïve^37^. Aposematism should, however, bring survival benefits to its carrier if local predators have learned to avoid the conspicuously signalling prey. The survival benefit is difficult to show in a natural setting, because predation events are rarely observed. Artificial model experiments are widely used to measure predation risk on prey phenotypes^38–40^, but attack rates towards models are often low and statistical power consequently may remain low as well. In the present experiment, we were not able to increase the sample size to the level that would have allowed power for detecting a statistically significant benefit of the signal. However, estimated attack risks provide suggestive support for aposematic function of the soles. The attack risk was lower for the red footed than non-red footed toads in the population where predators were familiar with the signal. In the population where red soles do not naturally exist, the attack risk was slightly higher for red footed than non-red footed toads.

The red feet of *Atelopus* appear similar to so-called flash marks, known from a range of animals such as grasshoppers, butterflies^3^, other amphibians^41^ or birds^42^. In the case of flash marks, the colour signal is not continuously visible and is used by diurnal and nocturnal animals for sexual signalling or predator avoidance. In birds, such flashing coloration is associated with sexual selection processes or with flocking behaviour, either to confuse predators or to coordinate flying^43^. In butterflies, flash marks are thought to confuse predators and in amphibians, similar to grasshoppers, flash marks or flash colours, have been associated with acute escape behaviour, deimatism and territoriality^41^. For example, the brilliant-thighed poison frog, *Allobates femoralis*, possesses inguinal red-orange to yellow colour patches that become visible when they make quick, long jumps during escape. This behaviour and type of colour signal can best be described by what Edmunds^3^ labelled “flash behaviour”. This behaviour falls in the context of deimatism; a ‘startle’ display that triggers a reflexive response in the predator to a sudden sensory input^44^. In the case of *Allobates femoralis* this works by irritating the attacker with a sudden colour signal, but hiding the colour and staying motionless after jumping away, consequently, inhibiting prey detection. Moreover, Toledo and Haddad^26^ describe flash colour behaviour in the nocturnal, arboricolous genera *Hypsiboas* and *Scinax*. Here, again, the colour signal is visible during movement but concealed during resting posture, protecting them from attacks during the day whilst resting. Both Toledo and Haddad^26^ as well as Hödl and Amézquita^41^ mention a potential aposematic function of flash colours.

The red feet of *A. spumarius* sensu lato differ from the examples above by being displayed independently from the presence of predators or conspecifics, and by being displayed by diurnal animals. Furthermore, other amphibians display flash colours for a short instance, whereas the red feet in *A. spumarius* sensu lato become visible repeatedly and during slow movement. This enables the predator to follow and process the signal, indicating a function other than confusion or short-term hesitation in order to escape. Moreover, both male and female *Atelopus* possess the signal where present. As with most members of the family Bufonidae, *Atelopus* males use scramble competition and multiple amplexi on one female have been documented^45^. Female mate choice is restrained in this type of mating competition and, therefore, the red feet are unlikely to be related to mate choice. However, we cannot completely exclude sexual selection in the context of interspecific communication purposes, for example in male-male interaction. Semaphoring and hand-waving could be observed in populations both with and without red feet with no obvious behavioural differences (own unpublished observations). Yet, it is possible that the signal serves multiple signalling functions, not only directed towards visually oriented predators but also towards conspecific males, which remains to be studied.

Being aposematic during movement, but less visible when at rest, may be beneficial as movement increases detectability by visually-oriented predators^46^. A similar function was identified for different larval stages of the alder moth (*Acronicta alni*). Early larval stages resemble bird droppings; thus, the animals use masquerade to avoid detection. However, in their last instar, when moving to their pupation sites, they change to a highly aposematic appearance^20^. The authors explain the change by the increased predation risk during active movement, thus, an aposematic strategy when moving would be beneficial. A study by Dimitrova *et al*.^47^ showed that high-contrast markings increase prey concealment on high-contrast backgrounds. Markings on the dorsum of the studied *Atelopus* are highly contrasting and are likely to function by breaking up the body outline, subsequently being a disruptive coloration, aiding concealment of the toad^21^. Furthermore, toads show high variation of patterns - from single spots to complex marbling - among individuals within one population, making it more difficult for potential predators to memorize and learn it. This indicates that the dorsal coloration may not have a strong interspecific signalling function, at least from a distance.

Some studies suggest that aposematism and crypsis are not as mutually exclusive as sometimes considered^48,49^. Since harlequin toads are small, it is questionable how visible the red feet are to avian predators when far away. A distance-dependent dual protection similar to that in the swallowtail butterfly (*Papilio machaon*) larvae studied by Tullberg *et al*.^19^ appears possible. Consequently, from a distance, the toads are protected by a dorsal pattern that blends in with the background. However, during close encounter, bright red feet could function as a strong warning signal, informing the predator of the prey’s toxicity. Indeed, it is possible that the dorsal pattern of harlequin toads has a similar quality: it is hard to detect from the distance but easy to recognize when a predator is close by. This hypothesis, however, needs further studies on distance dependent detection risk.

Our results suggest that red feet in the studied toads are an effective warning signal. The question remains, why they are lacking in other populations. Two potential explanations are worth considering. First, according to our behavioural data it is possible that we are dealing with two different strategies that have evolved in order to avoid predation. Toads without red feet may rely more on their overall cryptic appearance and behave less boldly by staying lower in the vegetation. It would be very interesting to test whether staying in lower vegetation actually increases survival in this species. The second explanation is offered by the predator mixes model by Endler and Mappes^50^. The model suggests weaker aposematic signals can evolve as a result of the constellation of a given predator community. Endler and Mappes argue that within a predator community “there is significant within- and among-species variation in search behaviour, in their visual, cognitive and learning abilities, and in their resistance to defences”^50^. Some studies indicate that prey can evolve weaker signals in order to remain better protected when co-occurring with several predator classes^28^. Comparing toads with and without red feet, the ones lacking the signal arguably display a weaker signal than the ones displaying red feet. Local predator communities in *A. spumarius* sensu lato populations might differ in their composition or abundance, driving the advantage or disadvantage of having red feet. Having red feet in a habitat with high predator abundance and diversity could attract attention resulting in increased predation risk, thus avoiding detection by means of cryptic coloration and less bold behaviour can be advantageous.

Lastly, when talking about evolving weaker or stronger signals one might expect differences in secondary defences. Consequently, we assume that toxin levels might differ between the studied populations. The interplay of conspicuous signals and toxicity has been subject to many different studies with conflicting results^51–53^. When dealing with honest signals which are supposed to benefit sender as well as receiver, more conspicuous signals should be positively correlated to higher toxin levels^54^. However, there is great variation in signal strength and signal honesty^55^ and even findings of inverse relationship between coloration and toxicity^56^. Consequently, it is not clear what the relationship between the signal of red hand and feet in the studied toads could be. Red footed toads being more toxic, less toxic or equally toxic to those lacking red feet are all possible outcomes when studying TTX levels in these populations. Moreover, it is important to state that TTX, being a different toxin than alkaloids found in poison dart frogs and being of unknown origin in this species, might have evolved in a different context. Thus, further studies are needed to understand toxicity in this genus. So far, however, accurate quantification of TTX levels would require euthanasia, which, in view of their endangered status, we do not find justifiable. As soon as accurate, non-invasive quantification is possible, we will analyse toxin levels of different populations and study how they correlate to coloration and behaviour of these toads.

## Conclusion

Protective coloration such as aposematic coloration in animals is an intriguing field of research that is still far from being completely understood. Red feet in harlequin toads present a fascinating example of how diverse and complex anti-predator strategies are. We introduce a comprehensive way of studying signal function by integrating different methods within one study, investigating different angles of potential signal mechanisms to understand its evolutionary function. To further understand the evolutionary function of the presence or absence of the signal, future studies should concentrate on the variability of the toxicity towards the predators, include more behavioural aspects, investigate the distance dependent detection risk, and the potential signal function of dorsal coloration.

## Methods

**Figure 5:**
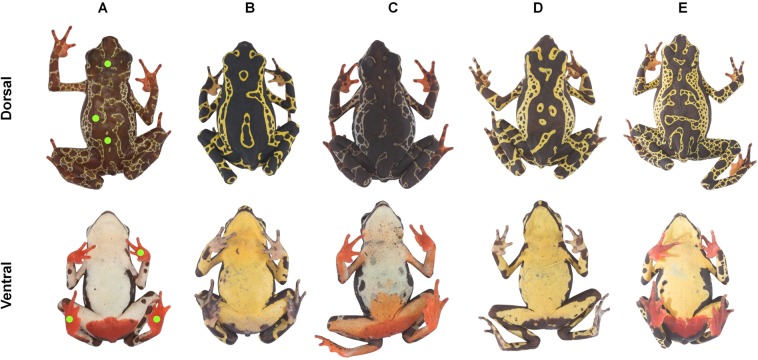
Dorsal and ventral view of five analysed harlequin toad populations from Brazil ((**A**) RFAD Campo Tinga, (**B**) REBIO Uatumã, (**C**) FLONA Tapajós, (**D**) SDN 1, (**E**) SDN 2), three with red feet, two not displaying additional colours. Green dots on left individual show points of spectrometric measurements of dorsal dark and red coloration. Photos: Matthijs P. van den Burg, Daniela C. Rößler.

Data were collected during the rainy season from March 1^st^ to May 1^st^ 2016 and from April 3^rd^ to June 3^rd^ 2017. We collected skin reflectance measurements from five *A. spumarius* sensu lato populations in Northern Brazil (for detailed localities see Supplementary Document S1) in order to obtain a large-scale sampling of red foot coloration (see Fig. 5). Foot reflectance spectra were obtained by measuring three points (left hand and both feet). For dorsal dark spectra, we measured three points of the dorsal dark colour from the head, dorsum and lower dorsum (see green dots in Fig. 5). All study sites were located in terra firme rainforests, characterized by a closed canopy and an understory vegetation with abundant sessile palms. In total, data on 52 male individuals were used for visual modelling calculations (Supplementary Table S1). All further experiments focused on the comparison between two populations (SDN1 and SDN2) only (see Fig. 5D,E). Detection experiments were carried out on a trail in a local rainforest close to the village of Serra do Navio in the state of Amapá. We measured the position heights of male individuals in two neighbouring populations (SDN 1 and SDN 2), one without, and the other with red feet (Supplementary Table S1) in order to test differences in behaviour. Clay model experiments were carried out on transects in each of these populations (more information on study species and data collection, see Supplementary Document S1).

### Visual modelling

For the collection of spectral reflectance data, we used a portable Ocean Optics HR2000 spectrometer with an Ocean Optics bifurcal optic fiber (R-200-2-UV/VIS) and an adaptor to ensure 2 mm distance between fibre and toad skin. The light source was a deuterium-tungsten DT-Mini-2GS lamp. As a white reflectance standard, we used the WS-1-SS, and calibration was carried out before every individual to account for lamp drift. Data collection was carried out with the software OceanView (Ocean Optics Inc.). Measurements were taken with the end of the fibre being placed on the toad’s skin surface in a 90° angle. Foot reflectance spectra were obtained by averaging three measurements, left hand and both feet. For dorsal spectra, we also averaged three measurements of the dorsal dark colour from the head, dorsum and lower dorsum. To keep the data comparable and to avoid bias caused by human viewer detection, we averaged a common foliage background based on 30 reflectance measurements from randomly collected leaves from the leaf litter for contrast calculations.

Irradiance was measured in each habitat at different times of the day and under different weather conditions with a 3900 µm optical fibre and a cosine adaptor held upwards. Three measurements were averaged for each locality and all measurements were averaged for a “standard common irradiance” that was used for the visual modelling calculations. We justify this with the high similarity of irradiance in rainforest habitats for all populations.

To evaluate the perception of the red feet for the visual systems of different predators, we used visual modelling following Vorobyev *et al*. to calculate both colour (∆S) and brightness (∆Q) discrimination for: bird (tetrachromatic), snake (trichromatic) and crab (dichromatic) vision^57–63^. The sensitivity information of cone classes for the studied predators are based on Hart^62^ for the avian visual system, Macedonia *et al*.^60^ for the snake visual system and Jordão *et al*.^59^ for a crab vision model. The modelled contrasts are the foot coloration against the common foliage background and the contrast between the foot coloration and the dorsal dark colour of the toad to account for different predator perspectives. A full description of the visual model calculations is found in the supplementary files).

We combined chromatic contrast data of all individuals with red foot coloration in one group (red) and the ones without distinct pigmentation of soles in another (non-red). The same was done for achromatic contrast data. As sample size and testing do not allow assumption of normally distributed data, we tested for significance of contrast difference between the two groups using two-tailed Mann-Whitney-*U* Test. For each predator we tested contrast of foot coloration to the background for both groups as well as contrast of foot to the dorsal dark and created boxplots to visualize the results.

### Detectability

As conspicuousness and perceptibility alone do not necessarily lead to detection, we conducted detectability experiments with 23 naïve human predators that were asked to search for toad models. We used three types of models: red footed, non-red footed and brown (control) clay models and placed them in a natural habitat. We set up a 50 m transect along a trail in a rainforest. On the transect we placed a total of 36 clay models: 12 with red feet, 12 without red feet and 12 cryptic, completely brown, models, which served as a control group and were predicted to be detected least. The position (1: leaf litter, 2: green leaf, 3: branch) as well as the site (left or right with a maximum distance of 1 m from the trail) on which the models were placed was randomized and equally distributed prior to the experiment. Each human predator was shown an example photo of a toad model (which resembled but not matched the types used in the experiment) prior to the start in order to standardize the idea of what to look out for. They had 5 minutes to slowly walk along the transect and point at a model whenever they detected one. All models were placed in a location visible from the trail, subjects did not have to move vegetation or bend down. Every detection was noted by a researcher walking directly behind the subject, further telling them to go slower or faster, in order to keep the speed at which the transect was walked comparable.

To analyse data of the detection experiment we used generalised linear mixed model (GLMM) with binomial distribution and logit link function. Fate of each toad model (detected or not detected) was included as response variable and it was explained by model type red soles, non-red soles and brown). To count for repeated measures within participant (several detection events) we included participant ID as random factor.

### Position heights

To collect behavioural data, we measured male position height in two neighbouring populations (SDN 1 and 2) in 2016 and 2017. In total we recorded heights of 57 red footed and 54 non-red footed toads. Heights were recorded at different times of the day to rule out bias due to weather conditions or activity times. Toads were encountered by vision, when they were calling or by accident when collecting data on the transects for the clay model experiment. Calling activity or number of calling toads did not differ between the populations. Thus, position height sampling was unbiased. When a toad was found, the height was measured using a laser distance meter (Floureon®) measuring the distance from the toad straight down to the ground. When the toad was found on the ground, the height was 0 cm. We analysed the position height data of the two groups using Mann-Whitney-*U* Test.

### Predation experiment

**Figure 6:**
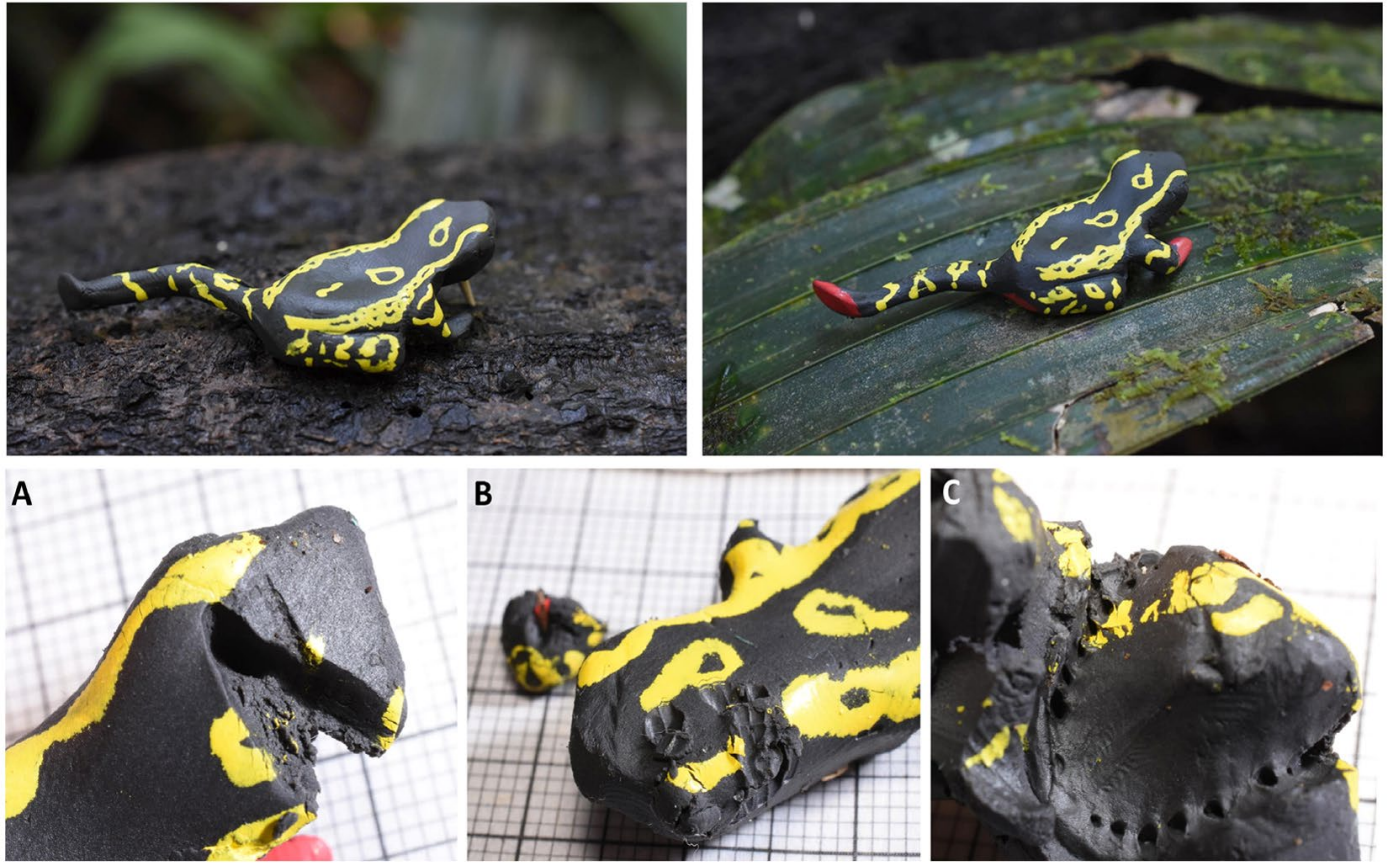
Types of clay models and attack marks. Top left: non-red footed model, top right: red footed model. Bottom: attack marks found on clay models. (**A**) avian beak mark, (**B**) rodent teeth mark, (**C**) lizard teeth mark.

To measure attack rates, we built clay models from nontoxic, black plastiline modeling clay (Noris Club®, Staedtler) and hand-painted the pattern and feet with nontoxic acrylic ink (AERO COLOR® Professional, Schmincke; colours: brilliant yellow and cadmium red hue). The colour of the clay as well as the ink on clay were measured prior to the field work to ensure reflection spectra resembled the spectral curve of toad coloration in that locality. Colours were shaded by adding brown acrylic ink until the spectra were similar. The models were made out of two-component silicone moulds. As the visibility of red feet is associated with movement, the posture of the clay models mimics a typical posture that the toads show during walking, with one leg and one arm stretched out (Fig. 6). The soles were painted red and a standard dorsal pattern was hand-painted on all models in yellow, which is the prevalent dorsal colour in the tested populations. The soles of non-red footed models were left black. Two 60 m transects were put in the forest (in SDN1 and SDN2) between June 3^rd^ 2017 and July 3^rd^ 2017, within one population of *A. spumarius* sensu lato with red feet and one population without red feet, always in close proximity and parallel to the local stream and in relatively dense vegetation. On each transect we placed 30 models with red feet and 30 models with non-red feet, each model type being placed on one of three possible positions (1: ground, 2: green leaf, 3: branch). All possible positions were equally distributed among model types and systematically randomized prior to the field work. Models were put every meter, alternating one meter to the left or right. Transects were checked every 72 hours for attacks. Attacked, destroyed or missing models were replaced by new ones in a randomized manner until the end of the experiment to avoid learning effects.

Attacked models were thoroughly inspected regarding attack marks and photographed. In cases of doubt, a neutral second rater was asked to confirm or discard attacks. In total we put 180 models on the transects, 83 in SDN1 and 97 in SDN2 (numbers are not equal due to replacements of missing and attacked models). For attack rates we only counted attacks by avian predators for each model and each transect. Although we detected attacks by rodents (see Fig. 6B), we decided to exclude them from the analyses as they are nocturnal. Attacks inflicted by insects, unidentifiable attacks as well as missing models were eliminated from further analysis. To evaluate the effect of foot coloration on predation risk we used generalized linear model (GLM) with binomial error distribution and logit link function. As the time that clay models were exposed to predation varied between individuals (due to replacing attacked and damaged clay models), we used attack risk within a day as a response variable and foot colour (red feet and non-red feet), population (naturally exhibiting red feet or not) and their interaction term as explanatory factors. This model was chosen because it best reflects hypothetical expectations of benefits of aposematism (i.e. increased attack risk when predators are naïve for the signal and decreased risk when the signal is learned). Model was fitted by using GLM function in *lme4* package in R^64^.

### Ethics

All procedures involving animals were conducted according to relevant legislation and by permission of the ethics committee of the Amazonas Federal University, CEUA/UFAM, number 039/2015. The research permit was issued by from Sisbio (Instituto Chico Mendes de Conservação da Biodiversidade (ICMBio) - Sistema de Autorização e Informação em Biodiversidade), number 45165-2.

## Supplementary information

Document S1

Table S1

Video S1

## Data Availability

All data supporting this article are provided in the main text or as part of the supplementary information. Additionally, the video can be found on YouTube under the following link: https://tinyurl.com/ycl7y2lv.
