# Author Correction: Sole coloration as an unusual aposematic signal in a Neotropical toad

**DOI:** 10.1038/s41598-019-55021-0

**Published:** 2019-12-06

**Authors:** Daniela C. Rößler, Stefan Lötters, Johanna Mappes, Janne K. Valkonen, Marcelo Menin, Albertina P. Lima, Heike Pröhl

**Affiliations:** 1 Department of Biogeography, Trier University, Universitätsring 15, 54296 Trier, Germany; 2 Centre of Excellence in Biological Interactions, Department of Biological and Environmental Science, University of Jyväskylä, P.O. Box 35, 40014 Jyväskylä, Finland; 3 Department of Biology, Institute of Biological Sciences, Amazonas Federal University, Av. General Rodrigo Otávio Jordão Ramos 3000, 69077-000 Manaus, Brazil; 4 Coordenação de Pesquisas em Biodiversidade, Instituto Nacional de Pesquisas da Amazônia, Av. André Araujo 2936, 69011–970 Manaus, Brazil; 5 Institute of Zoology, University of Veterinary Medicine Hannover, Bünteweg 17, 30559 Hannover, Germany

Correction to: *Scientific Reports* 10.1038/s41598-018-37705-1, published online 04 February 2019

This Article contains errors in the Results section under subheading ‘Visual Modelling’.

“For snakes, the visual model did not reveal any significance in terms of chromaticity between red and non-red feet. But, in terms of achromatic information, for the snake the contrast of red feet (11.10 ± 7.94 JND, N = 32) was significantly higher than the contrast of non-red feet (20.86 ± 10.89 JND, N = 20) against foliage background (U = 149, p < 0.001 two-tailed). Against dorsal dark coloration, the achromatic contrast of red feet (11.10 ± 7.94 JND, N = 32) was significantly smaller than for non-red feet (20.86 ± 10.89 JND, N = 20) (U = 148, p < 0.001 two-tailed).’’

should read:

“For snakes, the visual model did not reveal any significance in terms of chromaticity between red and non-red feet. But, in terms of achromatic information, for the snake the contrast of red feet (25.92 ± 7.97, N = 32) was significantly higher than the contrast of non-red feet (18.43 ± 9.29, N = 20) against foliage background (U = 149, p < 0.001 two-tailed). Against dorsal dark coloration, the achromatic contrast of red feet (11.10 ± 7.94 JND, N = 32) was significantly smaller than for non-red feet (20.86 ± 10.89 JND, N = 20) (U = 148, p < 0.001 two-tailed).’’

