# Supplementary material for: Sole coloration as an unusual aposematic signal in a Neotropical toad
Source: Sci Rep. 2019 Feb 4;9:1128. doi: 10.1038/s41598-018-37705-1 (PMC6362010; doi:10.1038/s41598-018-37705-1)
Supplement: Supplementary file 1 — Document S1 [file 41598_2018_37705_MOESM1_ESM.docx]

**Supplementary material, document S1 -**

**Supplementary methods for**

**Sole coloration as an unusual aposematic signal in a neotropical toad**

Daniela C. Rößler^1^*, Stefan Lötters^1^, Johanna Mappes^2^, Janne K. Valkonen^2^, Marcelo Menin^3^, Albertina P. Lima^4^, Heike Pröhl^5^

1. **Study Species**

The Amazon basin is inhabited by a monophyletic lineage of closely related forms of *Atelopus*^1^. The name *A. spumarius* Cope, 1871 is available for this lineage. Lötters et al.^2^ referred to this group as *A. spumarius* sensu lato since it potentially comprises a complex of species. Because the taxonomic relationship among Amazonian *Atelopus* remains unsolved we prefer to apply the name *A. spumarius* sensu lato for the toads we studied.

The absence or presence of red feet within the distribution of the species complex in Amazonian Brazil was unknown. We found five populations, in three the trait was present, while in the remaining two populations it was lacking. In the state of Amapá in Northern Brazil we found a unique setting. There are two populations of *A. spumarius*, sensu lato separated by the Amaparí River and approximately 11 km airline. In one population red feet are present whereas they are completely absent in the other. The close proximity of these populations gave us the opportunity to test several mechanisms linked to aposematism in order to gain insight into the function of red feet in this genus.

1. **Data collection**

Data were collected from five *A. spumarius* sensu lato populations in Brazil: Reserva Florestal Adolpho Ducke (RFAD), Campo Tinga (-2.9219500, -59.8982167); Reserva Biológica do Uatumã (REBIO Uatumã) (-1.8067667, -59.2655333); Floresta Nacional do Tapajós (FLONA Tapajós) (-3.0510333, -54.9685000); Serra do Navio, study site 1 (0.9425833, -51.9438167); Serra do Navio study site 2 (0.88904, -52.02398). Additionally in 2017 we found twelve individuals 3 km from the locality Serra do Navio 1, without red feet, located at the same stream (0.9425833, -51.9438167), allowing their assigning to Serra do Navio 1.

**Irradiance measurements**

Irradiance was measured in each habitat at different times of the day and under different weather conditions with a 3900 µm optical fiber and a cosine adaptor held upwards. Three measurements were averaged for each locality and all measurements were averaged for a “standard common irradiance” that was then used for the visual modelling calculations. Irradiance was measured in µW/cm² and was converted into Photon Flux (µmol/s/m²) for further calculations.

1. **Visual modelling**

Although the predator species that the models are based on do not occur in sympatry with *Atelopus*, we assume similarity of the visual systems between *C. caeruleus* and tropical bird species, the crab genus *Uca* and the crab genus *Fredius*, common in streams in Amazonia, as well as between *Thamnophis* and Colubridae/Dipsadidae snakes found in the Amazonian rainforest.

Prior to modelling, the reflectance spectra were interpolated in 1 nm steps from 300 nm to 700 nm as well as smoothed via triangular smoothing using the respective functions in AVICOL^3^. To model color and brightness discrimination, first the quantum catch for each cone (i) (for birds: UVS, SWS, MWS, LWS; for snakes: UVS, SWS, LWS; for crabs: SWS, LWS) was calculated taking into account receptor sensitivity spectrum R$\left( \lambda\right)$, background reflectance spectrum S$\left( \lambda\right)$ as well as irradiance spectrum I$\left( \lambda\right)$ for each wavelength between 300 and 700 nm $\left( \lambda\right)$ with equation 1.

| $Q_{i}= \int_{300}^{700} R_{i}\left( \lambda\right)S(\lambda)I\left( \lambda\right)d(\lambda)$ | (1) |
| --- | --- |

The quantum catches are then used to find a relative contrast for each receptor type *i*, in this case the contrast between (1) the feet and the background and (2) the feet and the dorsal dark coloration. The relative contrast of a receptor ($\Delta f_{i}$) is calculated by division of the natural log of the quantum catch of the two colours as in equation 2.

| $\Delta f_{i}= \frac{\ln Q_{i color 1}}{\ln Q_{i color 2}}$ | (2) |
| --- | --- |

To calculate discrimination for each receptor class, each receptor class is assigned a limiting Weber fraction,$\omega_{i}$, based on the noise-to-signal ratio of each cone, $v_{i}$, and the relative number of cones in each class (proportions),$\eta_{i}$ (see equation 3). Photoreceptor noise estimates used for measurements are: $v$ = 0.05 for birds^4^ as well as for snakes (following Maan and Cummings^5^); for crabs we used measurements of LWS cone class from the honeybee (*Apis mellifera*) as our measurement of photoreceptor noise $v$ = 0.12, following Cummings et al.^6^. Cone ratios were collected from the literature and used as follows: birds – UVS 1: SWS 1.9: MWS 2.2: LWS 2.1, VS model following Hastad et al.^4^; snakes – UVS 1: SWS 2: LWS 17 following information on the garter snake (*Thamnophis sirtalis*) by Sillmann et al.^7^; crabs – SWS 1: LWS 1 as in Cummings et al.^6^.

| $\omega_{i}=\frac{v_{i}}{\sqrt{\eta_{i}}}$ | (3) |
| --- | --- |

Based on all of the above calculations we then calculated colour contrast (∆S) (as defined in Vorobyev et al.^8^), expressed in JND units (just noticeable difference), for di-, tri- and tetrachromatic vision respectively with the following equations:

| $\left( {\Delta S}_{di} \right)^{2}= \frac{{(\Delta f_{1}-\Delta f_{2})}^{2}}{{\omega_{1}}^{2}+{\omega_{2}}^{2}}$ | (4) |
| --- | --- |
| $\left( {\Delta S}_{tri} \right)^{2}= \frac{{\omega_{1}}^{2}(\left( \Delta f_{3}-\Delta f_{2} \right)^{2}+{\omega_{2}}^{2}\left( \Delta f_{3}-\Delta f_{1} \right)^{2}+{\omega_{3}}^{2}{(\Delta f_{1}-\Delta f_{2})}^{2}}{\left( \omega_{1}\omega_{2} \right)^{2}{+\left( \omega_{1}\omega_{3} \right)}^{2}+\left( \omega_{2}\omega_{3} \right)^{2}}$ | (5) |
| $\left( {\Delta S}_{tetra} \right)^{2}=\frac{\left( \omega_{1}\omega_{2} \right)^{2}\left( \Delta f_{4}-\Delta f_{3} \right)^{2}+\left( \omega_{1}\omega_{32} \right)^{2}\left( \Delta f_{4}-\Delta f_{2} \right)^{2}+\left( \omega_{1}\omega_{4} \right)^{2}\left( \Delta f_{3}-\Delta f_{2} \right)^{2}+\left( \omega_{2}\omega_{4} \right)^{2}\left( \Delta f_{3}-\Delta f_{1} \right)^{2}{+\left( \omega_{3}\omega_{4} \right)}^{2}\left( \Delta f_{2}-\Delta f_{1} \right)^{2}}{(\left( \omega_{1}\omega_{2}\omega_{3} \right)^{2}{+\left( \omega_{1}\omega_{2}\omega_{4} \right)}^{2}+\left( \omega_{1}\omega_{3}\omega_{4} \right)^{2}+\left( \omega_{2}\omega_{3}\omega_{4} \right)^{2})}$ | (6) |

Brightness contrast (∆Q) was calculated under the assumption that only the LWS pigment is involved in brightness discrimination^9^. Equations 7-9 were used for tetra-, tri- and dichromatic vision, respectively:

| $\Delta Q_{tetra}= \frac{\Delta f_{4}}{\omega}$ | (7) |
| --- | --- |
| $\Delta Q_{tri}= \frac{\Delta f_{3}}{\omega}$ | (8) |
| $\Delta Q_{di}= \frac{\Delta f_{2}}{\omega}$ | (9) |

**Further supplementary information**

**Table S1.** Data (Visual modelling, position height data, positions of detection, predation data)

**Video S1.** *Atelopus* walking

**References**

1. Lötters, S. *et al.* Assessing the molecular phylogeny of a near extinct group of vertebrates: the Neotropical harlequin frogs (Bufonidae; *Atelopus*). *Syst. Biodivers.* **9,** 45–57, [https://doi.org/10.1080/14772000.2011.557403](https://doi.org/10.1080/14772000.2011.557403%20) (2011).

2. Lötters, S., Haas, W., Schick, S. & Böhme, W. On the systematics of the harlequin frogs (Amphibia: Bufonidae: *Atelopus*) from Amazonia. I: Description of a new species from the Cordillera Azul, Peru. *Salamandra* **38,** 95–104, (2002).

3. Gomez, D. *AVICOL. a program to analyse spectrometric data* (2006).

4. Hastad, O., Victorsson, J. & Ödeen, A. Differences in color vision make passerines less conspicuous in the eyes of their predators. *Proc. Natl. Acad. Sci. U.S.A*. **102,** 6391–6394, <https://doi.org/10.1073/pnas.0409228102> (2005).

5. Maan, M. E. & Cummings, M. E. Poison Frog Colors Are Honest Signals of Toxicity, Particularly for Bird Predators. *Am. Nat.t* **179,** E1–E14, <https://doi.org/10.1086/663197> (2012).

6. Cummings, M. E., Jordão, J. M., Cronin, T. W. & Oliveira, R. F. Visual ecology of the fiddler crab, *Uca tangeri*: effects of sex, viewer and background on conspicuousness. *Anim.Behav.* **75,** 175–188, <https://doi.org/10.1016/j.anbehav.2007.04.016> (2008).

7. Sillmann, A. J., Govardovski, V. I., Röhlich, P., Southard, J. A. & Loew, E. R. The photoreceptors and visual pigments of the garter snake (*Thamnophis sirtalis*): a microspectrophotometric, scanning electron miscroscope and immunisytochemical study. *J. Comp. Physiol. A* **181,** 89–101, <https://doi.org/10.1007/s003590050096> (1997).

8. Vorobyev, M. & Osorio, D. Receptor noise as a determinant of colour thresholds. *P. Roy. Soc. B-Biol. Sci.* **265,** 351–358, <https://doi.org/10.1098/rspb.1998.0302> (1998).

9. Siddiqi, A., Cronin, T. W., Loew, E. R., Vorobyev, M. & Summers, K. Interspecific and intraspecific views of color signals in the strawberry poison frog *Dendrobates pumilio*. *J. Exp. Biol.* **207,** 2471–2485, <https://doi.org/10.1242/jeb.01047> (2004).
